# The Medical and Surgical History of the War of the Rebellion

**Published:** 1888-09

**Authors:** 


**The Medical and Surgical History of the War of the Rebellion,** Part III., Volume I., Medical History; being the third medical volume. Prepared under the direction of the Surgeon-General United States Army. By CHARLES SMART, Major and Surgeon U. S. A. Washington: Government Printing Office. 1888.

This work is too extensive for a critical review. It is the third part of the medical history of the war, the first and second parts having been issued in 1870 and 1879 respectively. The volume considers the malarial and continued fevers; the eruptive fevers: (1) small-pox, (2) measles, (3) scarlet fever, (4) erysipelas. Other miasmatic fevers: (1) mumps, (2) yellow fever, (3) scurvy. Diseases of the respiratory organs: (1) catarrh, (2) epidemic catarrh, (3) acute bronchitis, (4) chronic bronchitis, (5)

asthma, (6) inflammation of the larynx, (7) inflammation of the tonsils, (8) diphtheritic inflammation of the fauces, (8) pneumonia, (9) pleurisy, (10) consumption. Rheumatic affections: (1) acute rheumatism, (2) chronic rheumatism. Other diseases attributed to exposure: (1) congestion and inflammation of the spinal membranes, ophthalmia, sunstroke. Cardiac diseases: morbid conditions, attributed to the weight of the accoutrements, constipation, headache, and neuralgia; jaundice, idiopathic peritonitis, diseases of kidneys. The volume concludes with Chapter XI., on certain diseases not heretofore discussed, and in which it treats of nostalgia, army itch, poisoning, alcoholism, venereal diseases. Chapter XII. takes up the consideration of general hospitals. We have given a brief epitome of the subjects considered in this valuable work with a view to present to our readers its scope and value. Surgeon Smart has performed a most important service to the profession in this compilation, and he has done his work well. The part is a valuable work in the library of the earnest student of medicine.

